# Association Between Clinical Signs and CBCT-Confirmed TMJ Involvement in Juvenile Idiopathic Arthritis: The Diagnostic Value of Facial Asymmetry and Mandibular Mobility

**DOI:** 10.3390/biomedicines14051164

**Published:** 2026-05-21

**Authors:** Tamara Pawlaczyk-Kamieńska, Tomasz Kulczyk

**Affiliations:** 1 Department of Dental Traumatology, Chair of Pediatric Dentistry, Poznan University of Medical Sciences, 61-701 Poznan, Poland; 2 Department of Diagnostics, Chair of Practical Clinical Dentistry, Poznan University of Medical Sciences, 61-701 Poznan, Poland; tkulczyk@ump.edu.pl

**Keywords:** juvenile idiopathic arthritis, temporomandibular joint, facial asymmetry, mandibular movements

## Abstract

Juvenile idiopathic arthritis (JIA) is the most common systemic chronic inflammatory connective tissue disease in children, characterized by joint inflammation lasting at least six months. Temporomandibular joint (TMJ) involvement can occur in conjunction with other joints and may often be asymptomatic in its early stages. **Objective**: This study aims to evaluate the relationship between clinical symptoms of the stomatognathic system and radiologically confirmed cone beam computed tomography (CBCT)-detected structural TMJ changes in children with JIA. The research hypothesis posits that specific clinical symptoms are more prevalent in patients with CBCT-confirmed structural TMJ changes. **Methods**: A cohort of children diagnosed with JIA was examined. Clinical symptoms, including facial asymmetry, limited mandibular movement, and joint and masticatory muscle pain upon palpation, were assessed. CBCT imaging was performed to assess osseous TMJ structural changes. **Results**: The frequency of orofacial clinical symptoms was assessed and compared between patients with and without radiological evidence of TMJ involvement. Children with CBCT-confirmed TMJ changes demonstrated significantly higher rates of facial asymmetry, reduced maximum mouth opening, mandibular deviation during opening, and limitations in lateral or protrusive movements compared with those without TMJ involvement. Pain-related symptoms (TMJ pain, muscle tenderness, and pain during movement) and joint sounds occurred at similar frequencies in both groups. **Conclusions**: Facial asymmetry, mandibular deviation during opening and reduced mandibular mobility are the clinical signs most strongly associated with structural TMJ involvement in JIA and should prompt targeted imaging. Pain-related symptoms show limited diagnostic value, highlighting the need for focused clinical assessment and future studies integrating CBCT and MRI to refine early screening protocols.

## 1. Introduction

Juvenile idiopathic arthritis (JIA) is the most common chronic inflammatory connective tissue disease in children, with onset before age 16 and symptoms lasting at least 6 months. It presents as oligoarticular (≤4 joints) or polyarticular (>4 joints), with oligoarticular JIA peaking between ages 1–3, and polyarticular between 2–4 and 6–12 years. Girls are affected twice as often as boys [1]. Temporomandibular joint (TMJ) involvement in JIA is common but frequently asymptomatic in early stages, earning it the label “forgotten joint” [2,3,4,5,6,7]. Inflammation in the TMJ may affect the condylar growth center, which plays a key role in mandibular development. Damage to this region can disrupt growth, leading to irreversible structural and functional changes in the joint [5,6,8,9,10].

Numerous studies have investigated the relationship between JIA and TMJ involvement; however, identifying early clinical changes in the TMJ remains challenging. Previous research highlights the importance of imaging techniques, such as MRI and cone beam computed tomography (CBCT), for diagnosis. Nonetheless, clear clinical criteria for early detection of inflammatory changes remain lacking. Understanding the relationship between facial asymmetry, limited jaw mobility, and radiologically confirmed structural TMJ changes may lead to the development of simple, reliable clinical indicators useful in everyday practice [5,8,9].

It is important to note that mild facial asymmetry and variations in jaw mobility can also be observed in healthy children. Liukkonen et al. (2005) [11] found that mandibular asymmetry may affect approximately 15–20% of healthy children. This underscores the need to distinguish physiological changes from those that are pathological and associated with JIA. Therefore, identifying reliable, easily detectable clinical indicators could facilitate early diagnosis of TMJ involvement and inform the need for imaging in patients with JIA.

Clinical consequences, such as facial asymmetry and restricted jaw movement, often emerge late and reflect the progression of chronic disease [4,6,9,10,12,13,14,15]. Distinguishing TMJ arthritis from temporomandibular disorders (TMDs) is essential, as they differ in aetiology, course, and treatment [16]. TMJ arthritis may be unilateral or bilateral and lead to joint dysfunction or impaired mandibular growth, depending on whether soft tissues or growth centers are involved. These changes are influenced by the mandible’s growth potential [4,7,14,15,17,18,19,20,21,22,23]. Early identification of TMJ involvement is crucial to prevent craniofacial deformities. As early symptoms are subtle or absent, reliable clinical indicators are needed to guide imaging and intervention before irreversible damage occurs.

This study aimed to assess the relationship between clinical symptoms of the stomatognathic system and TMJ involvement confirmed by CBCT in JIA children. The hypothesis was that symptoms such as facial asymmetry, limited mandibular mobility, midline deviation, and restricted jaw movements occur more frequently in patients with radiological evidence of structural TMJ changes.

Specific objectives:

1. Assess the frequency of key clinical symptoms (facial asymmetry, limited jaw mobility, and pain on palpation) in children with JIA.
2. Compare symptom patterns between patients with and without TMJ changes on CBCT.
3. Examine correlations between CBCT findings and mandibular function.
4. Identify clinical signs associated with CBCT-confirmed TMJ changes.

## 2. Materials and Methods

The present study was designed as a cross-sectional observational study conducted at the Department of Pediatric Dentistry, Poznan University of Medical Sciences. The study involved patients diagnosed with JIA according to the International League of Associations for Rheumatology (ILAR) criteria [1]. In the present study, all patients were diagnosed by pediatric rheumatologists, and the diagnoses were confirmed prior to their inclusion. Exclusion criteria included: systemic diseases unrelated to JIA, history of craniofacial trauma, and prior craniofacial surgery. The sample size was determined by the availability of eligible JIA patients meeting the inclusion criteria during the study period. Given the exploratory nature of the study and the limited patient population, no formal power calculation was performed. The findings of the study should be viewed as exploratory, intended to generate hypotheses rather than to confirm existing theories. The study did not include a separate healthy control group. Instead, comparisons were made between JIA patients with CBCT-confirmed TMJ changes (CBCT-positive patients) and those without such changes (CBCT-negative patients). Informed written consent was obtained from all participants and their legal guardians. Disease-related data were collected from rheumatology clinic records. An aggressive disease course was defined as rapidly progressive polyarticular disease requiring escalation to biologic therapy and/or persistent high inflammatory activity. The study was approved by the Ethics Committee of Poznan University of Medical Sciences (No. 1255/18).

Clinical examinations were conducted in a dental setting by a single examiner, while CBCT scans were independently evaluated by a second dentist with experience in radiological diagnostics of the stomatognathic system. Both were blinded to each other’s findings to reduce bias. A calibration protocol preceded data collection. The reliability of assessments was confirmed using Cohen’s kappa (categorical variables, e.g., facial asymmetry, joint pain): 0.78 (substantial agreement). Intraclass correlation coefficient (ICC) for continuous variables (e.g., range of motion): 0.86 (excellent agreement), Kappa for radiological assessment: 0.81.

Clinical examination included:

1. Facial symmetry was assessed using en-face photographs. Reference points (e.g., trichion, ophryon, pronasale, subnasale, stomion, gnathion) (Table 1) were marked to establish the facial midline and compare right and left sides [24].

2. Mandibular mobility was evaluated for:
  - Maximum mouth opening (MMO): measured between upper and lower incisors. A reduced range was <35 mm for children <10 years and <40 mm for those ≥10 years [25].
  - Deviation during mouth opening: evaluated using pencil lines marked on the lower incisors in central occlusion and at maximum opening. Deviations ≥ 2 mm were considered significant.
  - Lateral movements: lines were drawn in central occlusion and at maximal lateral displacement; reduced range was <6 mm (<10 years) or <7 mm (≥10 years).
  - Protrusion: measured between upper and lower incisors at full protrusion. Values < 3 mm (<10 years) or <4 mm (≥10 years) indicated reduction. All measurements were taken twice and averaged.
3. Muscle palpation included the masseter, temporalis, and medial pterygoid muscles. The lateral pterygoid was excluded due to limited palpation reliability.
4. TMJ examination involved palpation, assessment of joint pain during jaw movements, and evaluation for joint sounds (e.g., clicking). The general term “joint sounds” was preferred over “crackles” to avoid diagnostic ambiguity.

TMJ pain and masticatory muscle pain were recorded as dichotomous variables (present/absent) based on patient reports. No visual analogue scale (VAS) was applied in this study, as the aim was to assess the presence of pain rather than its intensity. When more than one masticatory muscle was painful on palpation, the finding was recorded as “muscle pain present,” without differentiating by muscle type, as the study focused on the presence of muscle tenderness rather than localisation.

Radiological assessment:

While MRI is the gold standard for detecting active inflammation in the TMJ, this study focused on the structural bone changes associated with chronic TMJ involvement. CBCT was chosen for its high spatial resolution, which is effective for assessing condylar morphology, and its availability at the study site. Routine MRI examinations were not possible during the study period due to the absence of a dedicated TMJ coil.

CBCT scans were performed with the CRANEX 3D system (Soredex, Tuusula, Finland) in the upright and habitual occlusal positions. Scans were obtained in a 16 cm × 13 cm FOV, with a voxel size of 0.2 mm, at 90 kV and 8 mA. Data were analyzed in OnDemand3D (Cybermed, Daejon, Republic of Korea). The condylar morphology was graded according to Billiau et al. [26]: 0—normal, 1—cortical erosions, 2—flattening, 3—flattening with erosions, 4—complete condylar loss. Patients were categorized into two groups based on CBCT findings:

1. those with radiological osseous TMJ changes (“CBCT-positive patients”),
2. those without such changes (“CBCT-negative patients”).

CBCT imaging was performed only when clinically indicated as part of routine diagnostic evaluation, in accordance with the ALARA principle, and not solely for research purposes.

Statistical analysis:

Data were analyzed using Statistica 12 (StatSoft, Tulsa, OK, USA). The Mann–Whitney test was used for comparing quantitative variables between groups. Categorical variables were analyzed with chi-square or Fisher’s exact tests, depending on sample sizes. Spearman’s rank correlation coefficient was used to assess relationships between variables. Results are presented as means ± SD, medians, and ranges. A *p*-value < 0.05 was considered statistically significant.

Sensitivity, specificity, positive predictive value (PPV), and negative predictive value (NPV) were calculated for each clinical parameter using classical 2 × 2 contingency tables. The following clinical variables were evaluated: facial asymmetry, deviation during mouth opening, limitation of MMO, lateral movement asymmetry, masticatory muscle pain, temporomandibular joint pain, and pain during jaw movements. Odds ratios (ORs) with 95% confidence intervals (CIs) were estimated for all clinical parameters in univariate analysis. Due to the presence of zero counts in some contingency table cells, the Haldane–Anscombe correction (adding 0.5 to each cell) was applied to ensure stable variance estimation and allow for the calculation of reliable confidence intervals. Multivariate analysis was not performed due to the exploratory nature of the study and the limited sample size. In addition, the low number of events per variable would substantially increase the risk of model overfitting and unstable estimates in multivariable modelling.

The study was reported in accordance with the STROBE guidelines for observational studies.

## 3. Results

A total of 40 patients with JIA, aged 6–17 years (mean 12.87 ± 3.39 years), were included. Girls represented 68% of the cohort. The mean age at JIA diagnosis was 9.92 ± 3.68 years, and the mean disease duration was 3.95 ± 2.39 years. An aggressive course was observed in 27.5% of participants, and the oligoarticular subtype in 47.5%. No significant demographic differences were found between CBCT-positive and CBCT-negative patients. CBCT identified TMJ changes in 57.5% of participants (20% unilateral, 37.5% bilateral).

Clinical signs, including facial asymmetry, reduced MMO, mandibular deviation during opening, and reduced lateral and/or protrusive movements, occurred significantly more frequently in CBCT-positive than in CBCT-negative patients (*p* < 0.05). Pain-related symptoms (TMJ palpation pain, pain during movement, masticatory muscle pain) and TMJ clicking occurred at similar frequencies in both groups (*p* ≥ 0.05) (Table 2).

**Figure 1:**
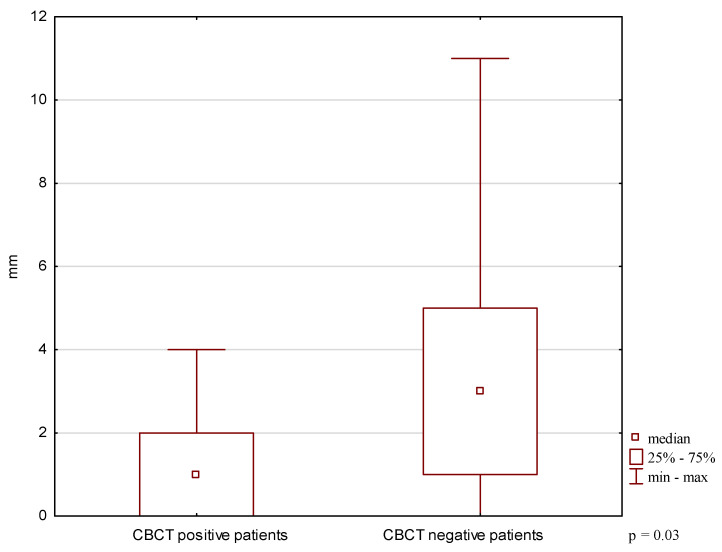
Asymmetry of lateral mandibular movements in patients with and without CBCT-confirmed TMJ changes.

**Figure 2:**
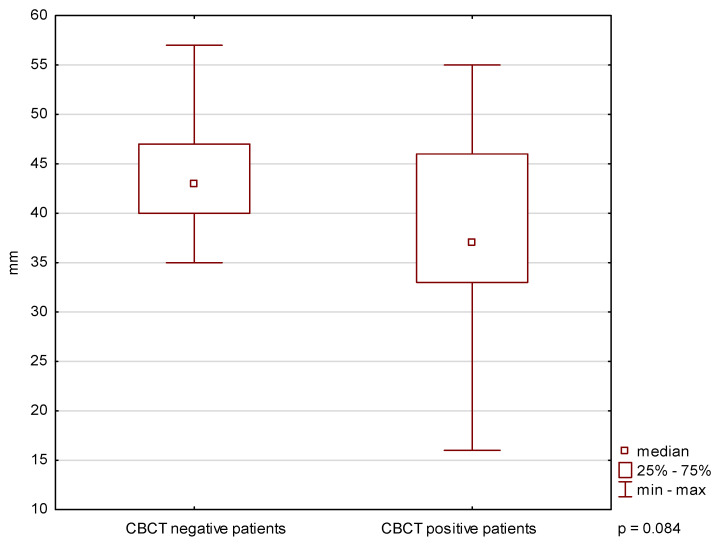
Maximum mouth opening in patients with and without CBCT-confirmed TMJ changes.

**Figure 3:**
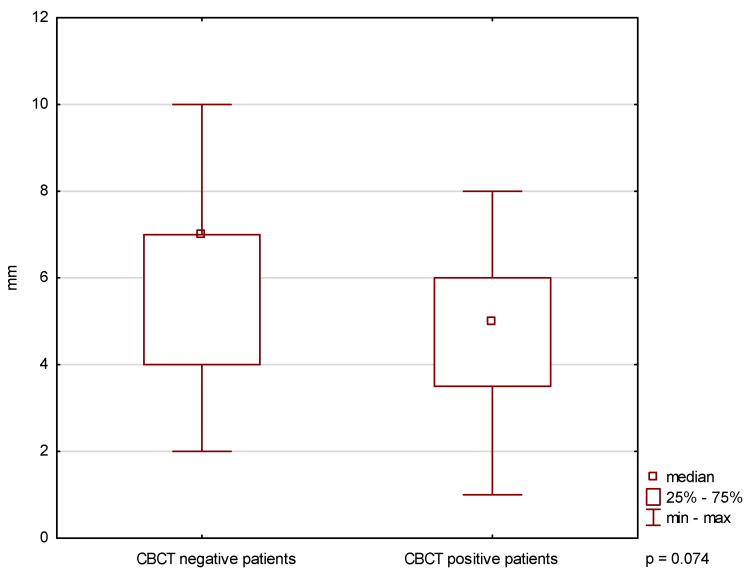
Maximum mandibular protrusion in patients with and without CBCT-confirmed TMJ changes.

A significant difference in the asymmetry of lateral mandibular movements was found between CBCT-positive and CBCT-negative patients (*p* < 0.05) (Figure 1). No significant differences were observed in MMO or protrusive movement range (*p* ≥ 0.05) (Figure 2 and Figure 3).

In the total JIA group, a significant negative correlation was found between age at diagnosis and reduced MMO. Multiple correlations were observed between clinical and functional variables. These correlations are presented in Table 3.

Mandibular deviation during opening showed a sensitivity of 43.5% and specificity of 100%. Facial asymmetry demonstrated a sensitivity of 26.1% and specificity of 94.1%. Reduced MMO showed a sensitivity of 52.2% and a specificity of 82.4%. Lateral movement asymmetry had a sensitivity of 78.3% and specificity of 29.4%. Pain-related symptoms showed sensitivity ranging from 8.7% to 26.1%, while specificity ranged from 64.7% to 82.4% (Table 4).

The ORs indicating the association between various clinical parameters and CBCT-confirmed TMJ pathology are as follows: mandibular deviation during opening: OR = 13.72 (95% CI: 1.57–119.89), facial asymmetry: OR = 6.29 (95% CI: 1.25–31.62), reduced MMO: OR = 4.09 (95% CI: 1.35–12.37), lateral movement asymmetry: OR = 1.50 (95% CI: 0.56–3.97). The wide confidence intervals reflect limited statistical precision and should be interpreted cautiously.

## 4. Discussion

Data in the literature regarding the frequency of TMJ arthritis during JIA are diverse, ranging from 17% to 87% [13,22,23,27,28,29]. Such a large range results from the applied TMJ examination methodology, as there are no uniform diagnostic criteria for the assessment of TMJ in JIA patients [24], and from the diverse characteristics of the population subjected to analysis (i.e., the age of patients on the day of examination, the age of patients in which the disease began, and the duration, course, and subtype of JIA) [5,30].

TMJ arthritis in JIA patients may affect one joint, although it is assumed to start in one joint and later involve the other, leading to more severe joint destruction [5,10,16,17,24,26,27,28,29,30,31]. Koos et al. [21], based on MRI, noted TMJ arthritis in 80% of JIA patients (unilateral in 25%). Weiss et al. [30] noted TMJ arthritis in 69% of examined JIA patients (32% unilateral). In the present study, based on CBCT, radiological changes were noted in 57.5% of the study participants, of which 37.5% were unilateral. In the remaining 20% of the study participants, the changes affected both joints, although the degree of their destruction was not always uniform. This may suggest that the disease first affects one TMJ, then the other. CBCT enables a precise analysis of the structure of each condylar process, thereby highlighting the differences between them [32]. However, it should be emphasized that radiological changes result from damage to bone structures and indicate a chronic process. Bakke et al. [33] demonstrated a relationship between the degree of change in the radiographic image of the TMJ and the duration of JIA. In the presented studies, the degree of mandibular deviation during mandibular abduction correlated with the degree of condylar damage, i.e., the mandible deviated towards the TMJ with a higher index value. Our findings are consistent with these observations, as mandibular deviation during opening in our cohort corresponded to the side with more advanced condylar damage on CBCT, supporting the concept of asymmetrical, progressive joint involvement.

The process of TMJ degeneration in the course of JIA is most often completely asymptomatic for a long time, with no pain or clinically or radiologically detectable abnormalities. The consequences of the diseases affecting the TMJ and possible damage to the condylar process and the mandibular growth center are usually observed several years after TMJ involvement. These consequences may include various dysfunctions of the masticatory system and possibly unilateral or bilateral mandibular underdevelopment, leading to dentofacial deformity [7]. In the initial stage of the disease, slight deviations from the normal state may be undetectable [4,24,34]. Spontaneous TMJ pain is not an indicator of TMJ arthritis in JIA patients. The literature data indicate that JIA patients complain more often of pain during jaw movements than spontaneous pain [4,7]. Carlsson et al. [35] reported this result in 6% of examined JIA patients, Weiss et al. [30] in 16%, Jank et al. [36] in 21%, and Zwir et al. [37] in 26% of examined JIA patients. In our study, pain during jaw movements affected 30% of CBCT-positive patients, with no statistically significant difference between CBCT-positive and CBCT-negative patients. According to these reports, our findings indicate that experiencing pain during jaw movements is common, but it does not serve as a distinguishing factor. This pain occurred with similar frequency in both CBCT-positive and CBCT-negative patients, highlighting its limited usefulness as an isolated clinical indicator of TMJ involvement.

There are also large discrepancies in TMJ palpation pain in JIA patients. Jank et al. [36] noted TMJ palpation pain in 23% of examined JIA patients, Assaf et al. [38] in 10%, Zwir et al. [37] in 33%, and Keller et al. [20] in 46%. In our study, TMJ palpation pain was noted in 12.5% of JIA patients, with no significant difference between the CBCT-positive and -negative patients. The results indicate that pain symptoms during jaw movements have limited diagnostic value for detecting temporomandibular joint involvement in the early stages of the disease. Koss et al. [21] also noted a significant difference between JIA patients and healthy peers in masticatory muscle pain (61% and 22%, respectively). Jank et al. [36] noted masticatory muscle pain in 20% of their study’s JIA patients, and Keller et al. [20] reported it in 47%. In our study, masticatory muscle pain was found in 20% of JIA participants and was similar in both groups. Regarding TMJ clicking, Koss et al. [21] reported a significant increase in masticatory muscle pain in JIA patients (22%) compared with the control group (12%). In our study, these symptoms were observed in 30% of JIA patients, with a similar rate in both groups. The combined findings show that there are no significant differences among the groups regarding TMJ palpation pain, masticatory muscle pain, and TMJ clicking. This indicates that these symptoms have low sensitivity and specificity for indicating structural issues with the TMJ. Therefore, they should not be relied upon as standalone screening criteria.

Notably, spontaneous pain, palpation pain in the TMJ or the muscles of the masticatory system, and pain during jaw movements are rather late symptoms of the ongoing inflammatory process, indicating its chronic nature [7,21,22]. The results of the studies confirm other authors’ reports of an asymptomatic or oligosymptomatic course of TMJ arthritis in JIA patients. Pain in the stomatognathic system is not a specific or characteristic marker of TMJ involvement [4,22,30,31].

A clinical symptom that may indicate CBCT-confirmed structural TMJ changes, particularly whether it affects one joint or is more severe in one of them, is facial asymmetry relative to the midline. This may result from inhibition of mandibular growth, which contributes to shortening the ramus on the side of the lesion and, consequently, the development of asymmetry of the lower facial segment [13,17,19,20,21,31,32,34,38,39,40,41]. In studies by Keller et al. [20], facial asymmetry, as assessed independently by a rheumatologist and an orthodontist, was noted in 37% and 41% of all JIA patients, respectively. At the same time, TMJ arthritis was diagnosed based on clinical examination and MRI in 71% of all patients, with 30% of these cases being unilateral [20]. In our study, facial asymmetry was noted in 25% of the examined patients, which was significantly more frequent in CBCT-positive patients than in CBCT-negative. As mild facial asymmetry and limited mandibular movement may also occur in healthy children, the absence of an age-matched control group limits the ability to distinguish JIA-specific alterations from normal developmental variability [11]. Future studies should include a healthy control group to strengthen the interpretability and clinical relevance of the findings. The findings therefore identify clinical markers associated with structural TMJ damage within the JIA population rather than markers specific to JIA compared with healthy children. In our analysis, we found that facial asymmetry demonstrated very high specificity and a significantly increased odds ratio for patients with CBCT-confirmed TMJ changes. This indicates that, although facial asymmetry is a relatively late manifestation, its presence strongly suggests underlying structural damage to the condyle. Therefore, it should prompt further imaging investigations.

Other symptoms of TMJ involvement may include limited MMO, limited range of lateral movements, uneven range of lateral movements to the right and left, and mandibular deviation during abduction [7,8,9,23,40,41]. Reduced MMO is clinically associated with possible TMJ involvement. Mohammed et al. [42] reported reduced MMO in 25% of JIA patients (80% of whom had TMJ arthritis). Similar results were obtained by Weiss et al. [30], who found a reduced range of MMO in 22% of JIA patients (69% of them diagnosed, based on MRI, with TMJ arthritis, of which 32% were unilateral; and based on US examination in 28%, of which 56% were unilateral). In contrast, Stoll et al. [13] noted reduced MMO (43% of patients with TMJ arthritis diagnosed by MRI) in 33% of patients with TMJ arthritis and 21% of patients without TMJ arthritis (this difference was not statistically significant). Our study used the MMO scale proposed by Müller et al. [25]. Using a 40 mm cut-off value in patients of developmental age may yield false-negative MO results, especially in children under 10 years, where MO > 40 mm is uncommon, even in healthy children. Our study showed a reduced MMO range in 40% of JIA patients, and significantly more often in CBCT-positive (57%) than in CBCT-negative patients (18%). Bakke et al. [33] demonstrated a relationship between the degree of change in the radiological image of the TMJ and the degree of reduced MMO. Our study did not confirm these correlations. However, reduced MMO in our material still showed moderate sensitivity and specificity for CBCT-confirmed structural TMJ changes, suggesting that while it is not directly proportional to the severity of condylar damage, it remains a clinically useful indicator of functional impairment associated with structural joint involvement.

Apart from facial asymmetry, a characteristic clinical symptom in CBCT-positive JIA patients may be mandibular deviation during abduction [4,8,9,17,18,19,20,21,22,32,34,38,39,40,41]. Jank et al. [36], Weiss et al. [30], and Hu et al. [39] reported jaw deviations during MMO in about 20% of patients with JIA. In contrast, a study by Stoll et al. [13] found that JIA patients with TMJ arthritis (49%) had significantly more jaw deviations than those without TMJ arthritis (12%). Koss et al. [21] demonstrated a significant difference in the percentage of JIA patients (62%) with jaw deviations during mandibular abduction compared to the control group (16%). In our study, jaw deviation during abduction was observed in 25% of JIA patients, and significantly more often in patients with CBCT-positive than in CBCT-negative patients. Moreover, our study has shown a relationship between differences in the degree of deformation of the right and left condylar processes on CBCT and differences in functional dysfunction of the masticatory system during lateral movements in CBCT-positive JIA patients. In addition, mandibular deviation during opening in our study presented perfect specificity and the strongest odds ratio among the analysed clinical parameters, indicating that when present, it may represent a clinically important indicator of asymmetric TMJ involvement and should prompt further diagnostic evaluation. The observed 100% specificity for mandibular deviation should be interpreted cautiously, given the small sample size and absence of false-positive observations in this cohort.

The degree of the resulting deformation is closely related to the growth potential of the mandible and depends on the child’s age when the disease begins. The most intensive growth of the mandible occurs in the period up to 6 years of age. In further development, lasting physiologically until the age of 21, the growth potential of the mandible decreases to 15% [4,7,23,33]. Kalaykova et al. [23] and Bakke et al. [33] showed that the younger the patient is at the time of JIA, the worse the TMJ prognosis, the more advanced the degeneration of TMJ structures, and the more advanced the joint dysfunction. Our study has shown that the younger the patient is at the time of diagnosis, the more frequently TMJ palpation pain is observed, and a reduced range of motion is noted. The development of morphological and functional abnormalities is also influenced by the time since the disease’s onset. These findings suggest that early-onset disease in our cohort is associated not only with structural risk but also with earlier functional compromise, supporting the need for particularly intensive TMJ surveillance in younger children at the time of JIA diagnosis.

In retrospective clinical and radiological studies of TMJ conducted among 94 patients twice (at the time of diagnosis and after 8.4 ± 5.8 years) by Kalaykova et al. [23], in the initial study, they did not observe any changes in the masticatory system in any patient, while in subsequent studies, long-term degeneration changes were observed in 82% of the examined patients. TMJ also has a worse prognosis in the polyarticular form [23,31,33]. Moreover, Kalaykova et al. [23] noted that the factors contributing to long-term TMJ degeneration (i.e., damage to joint structures, disturbance of mandibular mobility, and disturbance of mandibular growth and development) are also clinical symptoms observed in early JIA patients in the form of certain physical limitations in mandibular mobility, such as reduced MMO.

It should be emphasized that many factors, including genetic and environmental factors, influence the development of the stomatognathic system. In JIA patients, arthritis-induced dentofacial deformity depends on other factors, in addition to the course of TMJ arthritis and damage to the growth center. It is also dependent on a complex of many factors that impair the development of the facial skeleton, such as degenerations and deformations, mechanical overloads, dysfunctions, and parafunction of the masticatory system, as well as factors compensating for the abnormal development of the dentofacial complex [7,23]. TMJ arthritis, through damage to the growth center, directly affects the inhibition of mandibular growth, including inhibition and underdevelopment of the condylar process, which in turn induces deformation of the TMJ and its dysfunction, leading to increased friction in the joint, mechanical stress, and excessive load on the joint surfaces. If pathological mechanisms exceed the adaptive capacity of joint structures, deformations and dysfunctions become permanent, leading to progressive dentofacial deformity. This is because the resulting morphological and functional abnormalities of the TMJ can contribute to further destruction of joint structures.

In the case of morphological joint damage due to TMJ arthritis, but with optimal functioning and proper loading of the TMJ after active inflammation has subsided, the development of the masticatory system may proceed without disruption (if the growth center has not been damaged) [7,17,23]. Therefore, TMJ arthritis does not always lead to facial deformities. Dental therapy for JIA patients aims to diagnose TMJ arthritis early and take preventive measures against the development of dentofacial deformities. For this purpose, periodic clinical control of the TMJ and morphometric assessment of the dentofacial system are necessary, as the initial TMJ arthritis symptoms are not characteristic. For maxillofacial abnormalities, consultation and possible implementation of orthodontic treatment may be necessary. Orofacial physical therapy also seems advisable. Moreover, stomatognathic dysfunctions in pediatric patients, including temporomandibular disorders, sleep bruxism, and untreated dental conditions, may negatively affect sleep quality, daily functioning, and overall quality of life, further emphasizing the importance of early interdisciplinary management [43]. Our diagnostic analysis, showing that facial asymmetry, mandibular deviation, reduced MMO, and lateral movement asymmetry are significantly associated with CBCT-confirmed TMJ changes, supports incorporating these clinical parameters into routine screening protocols and highlights the need for close interdisciplinary follow-up.

Due to the retrospective nature of the available treatment data and incomplete medical documentation, pharmacological variables—such as the use of disease-modifying antirheumatic drugs (DMARDs), glucocorticosteroids, non-steroidal anti-inflammatory drugs (NSAIDs), and biologic agents—were excluded from the analysis. This constitutes a significant limitation, as these medications may affect both the clinical manifestations and radiological presentations of the temporomandibular joints. Future studies should incorporate standardized treatment-related variables into multivariate analyses to better control for potential therapeutic confounding factors. Moreover, unilateral and bilateral TMJ involvement may differ substantially in their clinical presentation and functional consequences. Due to the limited sample size, subgroup analyses based on the laterality of TMJ involvement could not be performed, which represents an additional limitation of the study. In this study, unilateral TMJ involvement was observed in 20% of patients, while bilateral involvement was observed in 37.5%. In our study, the sample size was limited by the number of patients who met the inclusion criteria during the study period and by the single-centre clinic. As a result, the statistical power for group comparisons was decreased, and the study should be considered exploratory. Furthermore, the single-center design and the regional character of the cohort may limit the generalizability of the findings to broader JIA populations with different ethnic, demographic, and geographic backgrounds. Therefore, the present results should be interpreted cautiously and considered exploratory until validated in larger multicenter studies. Additionally, the lack of an age-matched healthy control group restricts our ability to differentiate JIA-related changes from normal developmental variability. Therefore, the findings require validation in larger, multicenter studies that include appropriate control populations.

One limitation of the study is that pain variables were recorded in a dichotomous manner, categorizing them as either present or absent. This approach restricts the statistical methods that can be applied and prevents the use of traditional correlational analyses. The use of dichotomous pain variables limited the ability to assess symptom severity and may have reduced sensitivity for detecting subtle clinical differences. Future research should consider utilizing validated quantitative scales, such as the VAS, to enable a more detailed assessment of the relationship between pain intensity, masticatory function, and radiological findings.

Another limitation of the present study is the absence of a standardized DC/TMD-based examination protocol. The clinical assessment focused primarily on identifying functional and morphological manifestations potentially associated with TMJ involvement in JIA, rather than on comprehensive classification of temporomandibular disorders. Consequently, direct comparison with studies using validated DC/TMD diagnostic criteria may be limited. Future studies should incorporate standardized DC/TMD protocols to improve diagnostic consistency and comparability between investigations.

Another important limitation concerns the imaging modality used. Consequently, CBCT findings should be interpreted as indicators of structural or chronic TMJ involvement rather than active inflammatory arthritis. While CBCT provides high-resolution visualisation of osseous morphology and chronic structural alterations, it cannot detect early inflammatory changes such as synovitis, joint effusion, or bone marrow oedema. MRI remains the gold standard for identifying these pre-radiological inflammatory features and for distinguishing active inflammatory changes from chronic structural degeneration. In the present study, MRI could not be performed due to the unavailability of a dedicated TMJ-specific coil at our institution during the study period, thereby precluding a reliable assessment of soft tissue. This technical constraint limited our ability to evaluate early inflammatory involvement. Furthermore, the cross-sectional design of the present study precludes assessment of temporal or causal relationships. Longitudinal follow-up studies are needed to evaluate the progression of TMJ involvement and to validate potential early clinical indicators. Nevertheless, despite these limitations, the consistent associations observed between specific clinical signs and radiological TMJ changes in our study provide meaningful evidence that systematic clinical examination, supported by appropriate imaging, can improve early detection and risk stratification of TMJ involvement in children with JIA. Taken together, future multicenter prospective studies with larger sample sizes, standardized data collection, and combined CBCT–MRI imaging protocols—including detailed therapeutic records—are warranted to better control for confounding factors and to confirm the observed associations.

## 5. Conclusions

1. Facial asymmetry, mandibular deviation during opening, reduced maximum mouth opening, and limitations or asymmetry of lateral movements were the clinical findings most strongly associated with CBCT-confirmed TMJ involvement in JIA. Pain-related symptoms demonstrated low sensitivity and limited diagnostic value; therefore, the absence of pain does not exclude TMJ pathology in these patients.
2. Among the evaluated clinical signs, facial asymmetry and deviation during mouth opening showed the highest specificity and odds ratios, indicating that these features, when present, may reflect established or asymmetrical structural TMJ changes. However, their absence does not exclude TMJ pathology.
3. To prevent long-term consequences of TMJ-related deformities, regular clinical monitoring of the TMJ is essential in children with JIA. When abnormalities are identified, timely therapeutic intervention should be initiated—ideally within an interdisciplinary care model involving rheumatologists, dentists, orthodontists, and physiotherapists.

## Figures and Tables

**Table 1:** Anatomical landmarks used in the study.

Landmark	Description
*Trichion*	The point on the hairline in the midline of the forehead
*Ophryon*	The point at the mid-plane of a line tangent to the upper limits of the eyebrows
*Pronasale*	The most protruded point of the apex of the nose is identified in a lateral view of the rest position of the head
*Subnasale*	The point at which the nasal septum merges, in the mid-sagittal plane, with the upper lip
*Stomion*	The median point of the mouth when the mouth is closed
*Gnathion*	The lowest median landmark on the lower border of the mandible
*Incision superius*	The incisal tip of the most labially placed maxillary incisor
*Incision inferius*	The incisal tip of the most labially placed mandibular incisor

**Table 2:** Orofacial symptoms in JIA-examined patients.

	All Examined Patients (%)	CBCT-Positive Patients (%)	CBCT-Nagative Patients (%)	*p* Value
Facial asymmetry	25	39.13	5.88	*p* < 0.05 *
Reduced MMO	40	56.52	17.65	*p* < 0.05 *
Deviation during mandibular abduction	25	43.48	0.00	*p* < 0.05 *
Decreased lateral movements	47.7	65.22	23.53	*p* < 0.05 *
Decreased protrusion movement	45	60.87	23.53	*p* < 0.05 *
Pain of the masticatory muscles	20	21.74	17.65	*p* ≥ 0.05
Pain during palpation of TMJ	12.5	4.35	17.65	*p* ≥ 0.05
TMJ pain during jaw movements	30	26.09	35.29	*p* ≥ 0.05
TMJ clicking during jaw movements	25	30.43	17.65	*p* ≥ 0.05

* Statistically significant.

**Table 3:** Correlations between clinical and functional signs and radiological findings.

Variables	Correlation Coefficient (r)	*p*-Value
Reduced MMO ↔ reduced lateral movements	0.58	<0.001
Reduced MMO ↔ reduced protrusion	0.42	0.006
Difference in condylar damage ↔ difference in lateral movement range	0.51	0.004

**Table 4:** Diagnostic Accuracy of Clinical Symptoms for Detecting CBCT-Confirmed TMJ Changes.

Clinical Symptom	True Positive	False Negative	False Positive	True Negative	Sensitivity	Specificity	PPV	NPV
Facial asymmetry	6	17	1	16	*0.26*	**0.94**	**0.86**	0.48
Deviation in mouth opening	10	13	0	17	0.43	**1.00**	**1.00**	0.57
Reduced MMO	12	11	3	14	0.52	0.82	0.80	0.56
Lateral movement asymmetry	18	5	12	5	**0.78**	0.29	0.60	0.50
Masticatory muscle pain	5	18	3	14	*0.22*	0.82	0.62	0.44
TMJ pain	2	21	3	14	*0.09*	0.82	0.40	0.40
Pain during mandibular movement	6	17	6	11	*0.26*	0.65	0.50	0.39

**Bold values** indicate clinical parameters with the highest diagnostic utility. *Italic values* indicate parameters with low diagnostic usefulness. PPV—Positive Predictive Value. NPV—Negative Predictive Value.

## Data Availability

Data are available on request from the corresponding author.
